# Semi-Automatic Signature-Based Segmentation Method for Quantification of Neuromelanin in Substantia Nigra

**DOI:** 10.3390/brainsci9120335

**Published:** 2019-11-22

**Authors:** Gašper Zupan, Dušan Šuput, Zvezdan Pirtošek, Andrej Vovk

**Affiliations:** 1 Faculty of Medicine, University of Ljubljana, Vrazov trg 2, 1000 Ljubljana, Slovenia; gasper.zupan@mf.uni-lj.si (G.Z.); zvezdan.pirtosek@kclj.si (Z.P.); andrej.vovk@mf.uni-lj.si (A.V.); 2 Department of Neurology, University Medical Center, Zaloška 2, 1000 Ljubljana, Slovenia

**Keywords:** neuromelanin, neuroimaging, brainstem, substantia nigra, segmentation, Parkinson’s disease

## Abstract

In Parkinson’s disease (PD), there is a reduction of neuromelanin (NM) in the substantia nigra (SN). Manual quantification of the NM volume in the SN is unpractical and time-consuming; therefore, we aimed to quantify NM in the SN with a novel semi-automatic segmentation method. Twenty patients with PD and twelve healthy subjects (HC) were included in this study. T1-weighted spectral pre-saturation with inversion recovery (SPIR) images were acquired on a 3T scanner. Manual and semi-automatic atlas-free local statistics signature-based segmentations measured the surface and volume of SN, respectively. Midbrain volume (MV) was calculated to normalize the data. Receiver operating characteristic (ROC) analysis was performed to determine the sensitivity and specificity of both methods. PD patients had significantly lower SN mean surface (37.7 ± 8.0 vs. 56.9 ± 6.6 mm^2^) and volume (235.1 ± 45.4 vs. 382.9 ± 100.5 mm^3^) than HC. After normalization with MV, the difference remained significant. For surface, sensitivity and specificity were 91.7 and 95 percent, respectively. For volume, sensitivity and specificity were 91.7 and 90 percent, respectively. Manual and semi-automatic segmentation methods of the SN reliably distinguished between PD patients and HC. ROC analysis shows the high sensitivity and specificity of both methods.

## 1. Introduction

Parkinson’s disease (PD) is a common neurodegenerative disease with a prevalence of 160/100,000 (1.6 percent) in Western Europe [1]. Degeneration of the nigrostriatal dopaminergic system results in characteristic pathological changes in the substantia nigra (SN), a midbrain area that is crucially involved in movement [2]. Clinical diagnosis of PD is sometimes challenging, even when performed by an experienced neurologist. A meta-analysis [3] reports that the accuracy of the initial clinical diagnosis of PD made by a movement disorders specialist is 79.6 percent. The current role of brain magnetic resonance imaging (MRI) in PD is the exclusion of cerebrovascular diseases or other lesions that could cause or be attributed to parkinsonian symptoms [4]. Positron emission tomography (PET) offers a reliable distinction between patients with Parkinsonian disorders and healthy subjects and can differentiate between PD and Parkinson plus syndromes [5,6,7]. Dopamine transporter single-photon emission computed tomography (DaT-SPECT) can reliably differentiate some, but not all, parkinsonian syndromes [8,9,10]. Moreover, both PET and SPECT emit X-rays [11].

Neuromelanin (NM) is found in the brainstem of the human central nervous system [12]. NM is produced from dopamine and other catecholamines via quinone reactions followed by polymerization [13]. It is present in catecholaminergic neurons in the SN and locus coeruleus. NM may be important as a defense against the neurotoxicity of quinones produced in the process of dopamine oxidation [14]. There is no clear consensus on whether the number of NM-containing neurons decreases with age; however, in patients with PD, the loss of pigmented neurons is abundant [15]. NM is a paramagnetic substance and can be visible as hyperintensity on T1-weighted magnetic resonance (MR) images [16]. In 1986, Duguid et al. [17] observed a reduced width of SN pars compacta in patients with PD using a 1.5T scanner. Since then, several MR studies report a decrease in signal intensity [18,19,20,21,22,23,24,25,26,27,28] and volume or surface reduction [19,21,22,25,26,28,29,30,31,32,33,34] of the SN in patients with PD. Moreover, discrimination between PD patients and other Parkinsonian syndromes also yields reliable results [29,35,36].

This study aimed to investigate surface and volume changes of the SN in PD. Manual volumetric analysis of the SN is time-consuming and subjective. Therefore, we applied a novel semi-automatic local statistics signature-based segmentation method [37,38] to quantify the volume of SN.

## 2. Materials and Methods

### 2.1. Subjects

The National Medical Ethics Committee approved this study. Written informed consent was obtained from each participant before their inclusion in the study. Twenty right-handed patients with idiopathic PD (5 females, average age: 67.1 ± 4.8) were included in the study. Patients were invited to participate during their regular visits to the outpatient department of The Division of Neurology. They reported no additional neurological or psychological complaints at the time of inclusion. Hoehn & Yahr score (HY) and Mini-Mental State Examination (MMSE) were performed in PD patients. Because this study aimed to investigate the early stages of PD, only patients with HY 2 and 2.5 (mild bilateral PD) were included.

Patients were previously diagnosed with PD by a neurologist specialized in movement disorders based on clinical assessment. Twelve healthy right-handed subjects (6 females, average age: 64.0 ± 4.8) were included as a control group (HC). Healthy subjects were invited via Internet advertisements on the institutional homepage. Any known neurological or psychological disease in HC was a basis for exclusion.

Moreover, MMSE was performed in HC as well. The Edinburgh Handedness Inventory was used to confirm dexterity in participants. Participants with an unclear clinical diagnosis of PD, any contraindication for magnetic resonance imaging (MRI), MMSE < 26 were excluded from the study. In this study, one PD patient was excluded due to the presence of an unverifiable metallic implant in the body.

### 2.2. MR Parameters and Study Protocol

Structural MR images were acquired on Philips Achieva 3TX (Best, The Netherlands). T1-weighted gradient echo and T2-weighted turbo spin-echo pulse sequences were performed. The following parameters were used in T1-weighted and T2-weighted images, respectively: echo time (TE): 5.9 ms/403 ms, repetition time (TR): 12 ms/2500 ms, flip angle: 8°/90°, acquisition time: 11 min 55 sec/6 min 58 sec. The matrix was 320 × 336, the field of view was 224 × 235 mm, the voxel dimension was 0.7 × 0.7 × 0.7 mm, the number of sagittal slices was 236, and the number of averages was 2.

The area of brainstem and cerebellum from mammillary bodies to the end of medulla oblongata was scanned using a T1-weighted spectral pre-saturation with inversion recovery (SPIR) sequence. The acquisition was positioned perpendicular to the floor of the fourth ventricle. SPIR sequence parameters were TE 2 ms, TR 25 ms, flip angle 20°, a field of view 220 × 185 × 60, voxel dimension 1 × 1 × 1 mm, number of slices 60, number of averages 1, and acquisition time 4 min 33 sec.

### 2.3. Segmentation of Brain and Brainstem

Cortical reconstruction and volumetric segmentation were performed by the Freesurfer image analysis suite 6.0 (Boston, MA, USA), which is documented and freely available for download online (http://surfer.nmr.mgh.harvard.edu). Brainstem segmentation was performed with Brainstem Substructures [39], a Freesurfer add-on, which is also freely available online. The technical details of these procedures were described in previous publications [40,41,42,43,44,45,46]. Automatic skull-striping usually produced some residuals from the surrounding structures. These were removed manually to ensure proper further data processing such as white and grey matter delineation.

### 2.4. Segmentation of Substantia Nigra, Surface and Volumetric Analysis

A semi-automated analysis was performed using local statistics signature-based segmentation, as described in a previous publication [37,47]. This is based on the fact that not all voxels of the same tissue type exhibit similar signatures. Brain gray matter has different signatures from the gray matter in the basal ganglia or the gray matter in the brain stem. Semi-automated segmentation and quantification were performed using AFNI (Bethesda, Maryland, USA), version 17.0.06, available online (afni.nimh.nih.gov). The first step of semi-automatic segmentation was the creation of 4D volume by combining local statistics computed on neighboring voxels captured with a sphere mask, with a radius from 1 to 5 mm, on each voxel. Those different statistics computed on the different neighborhoods on each voxel represented the 4th dimension of 3D volume and were called signatures. Afterward, the signature inside the SN region was selected from the 4D volume in the AFNI Graphical user interface (GUI), and its correlation to other signatures was computed on the fly. In order to limit the correlation mask only to SN, the volume mask threshold was selected using the computed correlation. This signature and threshold selection steps were repeated a few times and added to the previous mask until the SN was fully covered. In the last step, a 3D model of brainstem and the SN was constructed in Blender (Amsterdam, The Netherlands), version 2.78 (www.blender.org).

**Figure 1:**
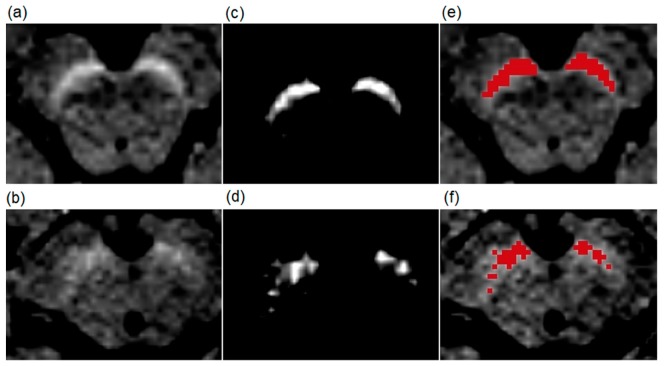
Examples of T1-weighted spectral pre-saturation with inversion recovery (SPIR) images of a patient (70-year-old male) with Parkinson’s disease and a healthy subject (73-year-old male). The upper row shows images from the healthy volunteer, and the lower row images from the patient. (**a**,**b**) are native T1-weighted SPIR magnetic resonance (MR) images. The results of the manual segmentation method are presented in (**c**,**d**). The bright areas are the left and right substantia nigra (SN) at the level of the highest hyperintensity. (**e**,**f**) show red-colored SN surface after the manual segmentation.

Manual delineation of the SN in mesencephalon was performed in 3DSlicer, version 4.9.0. (www.slicer.org). The examiner was instructed to adjust the contrast and brightness to completely exclude all the background information except the hyperintense areas of NM in the SN (Figure 1, central column). In the next step, the segmented hyperintense signal was quantified as the level of highest hyperintensity (Figure 1, right column). In case of doubt, the signal was quantified at more levels, and the largest surface area was included in further analysis. The status of a participant was not known to researchers in order to exclude observer bias in image analysis.

**Figure 2:**
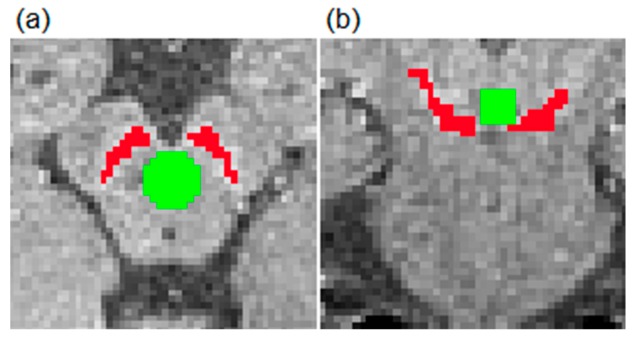
Presentation of areas for signal intensity (SI) analysis in the transverse (**a**) and coronal (**b**) planes. SI_SN_ and SI_background_ were defined as shown with red and green colors, respectively. SN: substantia nigra.

Moreover, signal intensity (SI) of the SN (SI_SN_) and the background (SI_background_) were also calculated in AFNI. SI_SN_ was acquired from the volume that was produced with a semi-automatic segmentation method. The SI_background_ was acquired from the sphere with a diameter of 4.9 mm. The sphere was positioned posterior to SN and anterior to the aqueduct at the level of highest SN hyperintensity, as shown in Figure 2.

SI_SN_ was normalized with SI_background_ to yield SI_norm_ with the following formula:

SI_norm_ = SI_SN_/SI_background_. (1)

In the last step, the results of surface and volume analysis were normalized (Surface_norm_ and Volume_norm_, respectively) with midbrain volume (MV). The following formulas were used:

Surface_norm_ = surface/MV × 1000 (2)

Volume_norm_ = volume/MV × 100. (3)

An analysis of acquired and blinded MR data was independently performed by two qualified researchers with 7 and 3 years of experience.

Q-Q plots were created to investigate the normal distribution of data. Independent-samples *t*-tests were used to explore differences between groups. Sensitivity and specificity were determined with a Receiver Operating Characteristic (ROC) curve analysis. Statistical analysis and graphic representation were performed with in-house IBM SPSS, version 25.0. (Armonk, NY, USA). The value of *p* < 0.05 was considered significant.

## 3. Results

At the time of MR data acquisition, the mean ages of patients and HC were 67.1 ± 4.8 years and 64.0 ± 4.8 years, respectively. The mean MMSE score was 29.8 ± 0.5 and 29.2 ± 1.1 in the PD group and HC group, respectively. The mean duration of PD was 3.7 ± 2.8, and the mean HY was 2.1 ± 0.2. Summarized demographic data of both groups are listed in Table 1.

**Figure 3:**
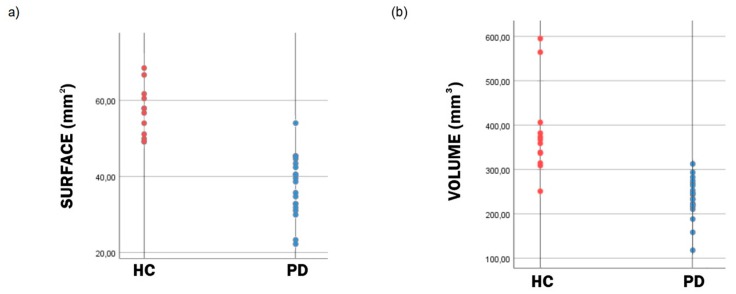
Scatter plots of the surface (**a**) and volume (**b**) of the segmented substantia nigra (SN). Distribution of the substantia nigra (SN) surface (**a**) and volume (**b**) data indicates that both the surface and volume of the SN is lower in patients with Parkinson’s disease (PD) in comparison to healthy controls (HC).

In patients with PD, compared to HC, the manual segmentation method produced, on average, a 33.7 percent lower surface of SN. The semi-automatic method also demonstrated a 38.6 percent lower mean volume of the SN in the PD group compared to HC. Both differences in surface and volume between groups were significant (*p* = 0.0001 and *p* = 0.002, respectively), indicating the adequacy of the semi-automatic method. The SI_norm_ of PD patients was 1.27 and for HC 1.28. There was no significant difference in mean SI_norm_ between patients with PD and HC (*p* = 0.861). Results of surface and volumetric analysis of the SN for all participants are presented in Figure 3, and data are summarized in Table 2.

Data on the surface area presented in Table 2 were obtained by manual segmentation of a slice taken from the maximal cross-section of the SN performed by two independent and experienced researchers. An example of manual segmentation is presented in Figure 1.

**Figure 4:**
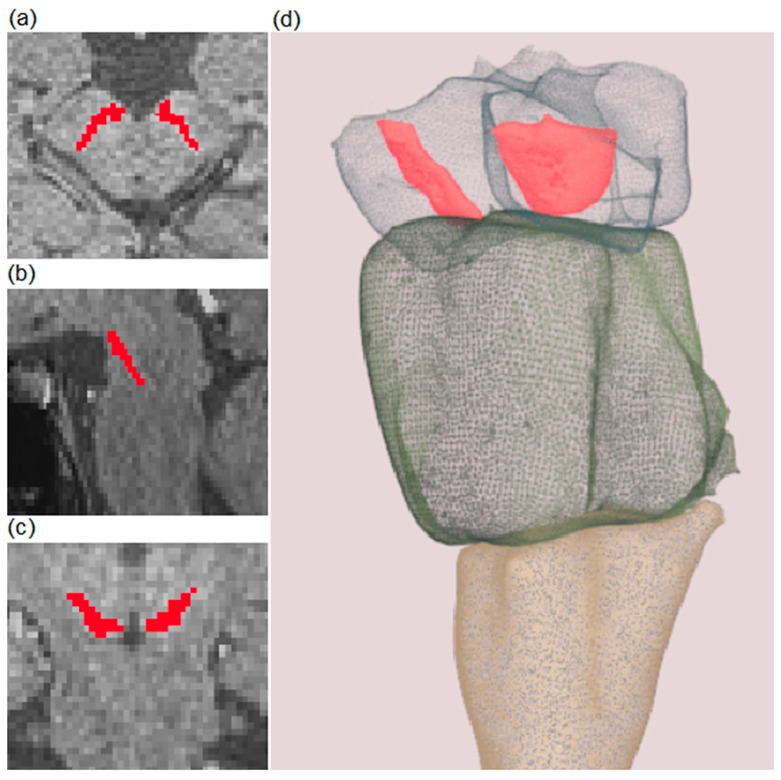
Example of semi-automatic segmentation of a substantia nigra (SN) on T1-weighted spectral pre-saturation with inversion recovery (SPIR) images. Red-colored areas represent segmented SN. Transverse, sagittal, and coronal planes are presented in (**a**–**c**), respectively. A 3D model of the brainstem (presented as a transparent structure for better visualization) and SN (colored in red) are shown in (**d**). A full example of the 3D model is included in Supplementary Files.

Although the amount of total volume reduction of SN in patients with PD is relevant, it is not readily evaluated in a clinical setting as the manual segmentation of all slices followed by calculation of the total SN volume is unpractical and time-consuming. An example of the semi-automatic segmentation method used to overcome those obstacles in this study is shown in Figure 4. A full example of the 3D model is available in Supplementary Files.

Results of the surface area and volume of SN were normalized with MV, which was produced in the process of brainstem segmentation. Segmented MV did not differ significantly between PD patients and HC. After normalization relative to the M, surface and volume remained significantly different between groups (*p* = 0.001 and *p* = 0.0004, respectively). Normalized results of volume and surface of SN are shown in Table 3.

**Figure 5:**
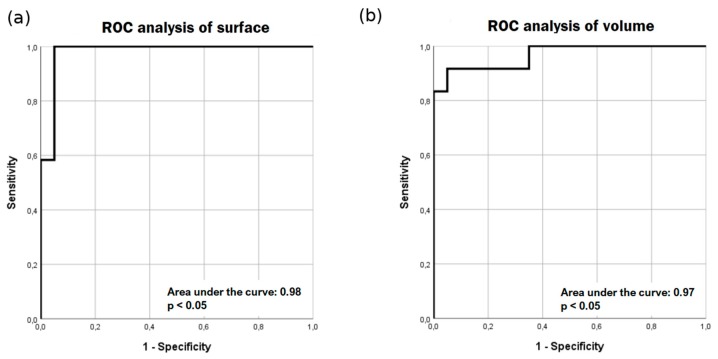
Receiver operating characteristic (ROC)analysis of surface (**a**) and volume (**b**) results. ROC analysis yields high sensitivity and specificity for manual segmentation of surface area (**a**) and semi-automatic (**b**) segmentation of volume.

ROC analysis was performed for surface and volume results (Figure 5). In the ROC analysis of the surface, at the cut-off value of 49.2 mm^2^, the sensitivity and specificity were 91.7 percent and 95 percent, respectively. In ROC analysis of volume, at the cut-off value of 287.9 mm^3^, the sensitivity and specificity were 91.7 percent and 90 percent, respectively.

## 4. Discussion

The SN is always affected by PD [2]. This study demonstrates a significant reduction in the surface and volume of the SN in patients with early PD compared to HC. A simple manual segmentation method yields a lower mean surface of the SN in patients with PD, which is true also after normalization of the results with MV. Several other papers also report a significant decrease in the surface of the SN [21,25,29,34]. Motor symptoms of PD appear when 30 percent or more neurons in the SN are destroyed [15]. However, in 2012, Oikawa et al. [48] showed no changes in the surface of the SN in patients and HC. They also observed a relatively substantial overlap between patients with PD and controls. The authors used a 1.5T scanner with an accordingly lower signal-to-noise ratio (SNR), which could lead to inaccurate detection of the SN. Most of the later studies were performed on 3T scanners to detect SN changes. Acquisition of images with 7T scanners offers even higher SNR and, therefore, better delineation of the SN [49]. However, the sensitivity and specificity of 3T scanners in discriminating alterations in SN volumes of patients with PD and HC are high, 70.8–100, and 72–100 percent [21,26,29,30,31,32], respectively. Our study demonstrates the high sensitivity and specificity of both segmentation methods as well, confirming the results of previous studies.

Threshold segmentation is the most commonly used method in the volumetric analysis of the SN. The methodology of data acquisition and analysis varies between studies, as segmentation can be performed at one or more levels in the mesencephalon. Moreover, the definition of cut-off threshold values varies as well. Our manual method involves manually adjusting the contrast to isolate only hyperintense areas of the SN and segmentation at one level. In our opinion, this is the simplest possible method and could be easily used in the clinical setting. Using manual delineation of the SN, studies reported [50,51,52] that the SN size correlated with DaT-SPECT results in PD. Okuzumi et al. [50] found that several clinical tools commonly used to assess the severity of PD correlated better with SN size but not as well with DaT-SPECT results. However, manual segmentation methods tend to be subjective, and volumetric analysis is unacceptably time-consuming. The semi-automatic method that we used [37] in this study produces tissue prior maps that are not affected by bias fields, and it does not require registration to a template space or the use of spatial priors. The priors provide a means for initializing and regularizing the segmentation model without adding the complication associated with optimizing parameters for spatial registration and bias field estimation. A voxel’s location in the priors’ space is used to gain information about its tissue type. However, our method acquires such information by examining the 3D texture of the image at that voxel location, and over multiple spatial scales as well.

Castellanos et al. [31] demonstrated a decrease in volume of the SN in patients with PD using automated segmentation with a reference atlas. After normalization of images, hyperintense voxels in SN masks were produced with thresholding. Similar results were demonstrated by Takahashi et al. [19] with an automated segmentation method utilizing a voxel-based morphometric technique. A semi-automatic method performed by Ogisu et al. [32] yielded a reduced volume of the SN in patients with PD as well. This semi-automatic method consisted of manually positioning “seed points” in the hyperintense area. After defining a level of threshold, the volume of interest was automatically produced. A similar method was used by Reimao et al. [34] as well.

We did not demonstrate any differences in SI_norm_ between the two groups; however, several studies show a decreased SI_SN_ in patients with PD compared to HC [18,19,20,21,22,23,24,25,26,27,28]. We might fail to demonstrate any difference in SI_norm_ due to the small sample size. Most of those studies used a T1-weighted fast spin-echo sequence similar to a sequence that was first described by Sasaki et al. [24]. Schwarz et al. [25] report a small yet significant difference in contrast ratios between PD patients and HC (1.15 vs. 1.18, respectively). It is important to note that studies showed a difference in SN SI and SN volume [29,35,36] between PD patients and patients with progressive supranuclear palsy, hence producing evidence that SN imaging might have a role in differentiation between some parkinsonian syndromes.

This study used a semi-automatic signature-based segmentation method that demonstrates volume reduction in patients with PD compared to HC. Moreover, after correcting the results with MV, the difference in volume between groups was observed as well. This finding is in agreement with the results from previous studies [19,26,28,30,31,32,33]. However, the process still has to be checked by the examiner to ensure high-quality analysis.

This study has limitations. The first limitation is a small sample. In the future, a multi-centric study with longitudinal design may be needed to assess the advantages and disadvantages of NM-sensitive MRI in an everyday clinical setting. The other limitation is the fact that our sample does not include PD patients with all grades of the disease.

## 5. Conclusions

In conclusion, we used a T1-weighted SPIR sequence to increase the contrast between white and grey matter. In previous papers, this sequence was rarely used for the study of Parkinson’s disease. Excellent demarcation of the SN was achieved in our study, as well. Manual and semi-automatic signature-based segmentation methods of the SN reliably distinguish between PD patients and HC. High sensitivity and specificity of both methods in the detection of decreased surface and volume of the SN in PD were found with ROC analysis. Moreover, the semi-automatic method allows for a reliable and straightforward assessment of the whole volume of the SN.

## Figures and Tables

**Table 1:** Demographic and clinical features of the sample.

	PD	HC
Number	20	12
Age	67.1 ± 4.8	64.0 ± 4.8
Duration of PD	3.7 ± 2.8	/
HY	2.1 ± 0.2	/
MMSE	29.8 ± 0.5	29.2 ± 1.1

PD: Parkinson’s disease; HC: healthy controls; HY: Hoehn & Yahr, MMSE: Mini-Mental State Examination.

**Table 2:** Surface, volume and signal intensity of the substantia nigra.

	PD	HC	Group Comparison
Surface (mm^2^)	37.7 ± 8.0	56.9 ± 6.6	*p* = 0.0001
Volume (mm^3^)	235.1 ± 45.4	382.9 ± 100.5	*p* = 0.002
SInorm	1.27 ± 0.04	1.28 ± 0.03	*p* = 0.861

PD: Parkinson’s disease; HC: healthy controls, SI_norm_ = normalized signal intensity.

**Table 3:** Normalized results of surface and volume of the substantia nigra.

	PD	HC	Group Comparison
MV (mm^3^)	6244.8 ± 650	6391.1 ± 864.3	*p* = 0.877
Surface/MV	6.1 ± 1.4	9.1 ± 1.7	*p* = 0.001
Volume/MV	3.8 ± 0.7	6.0 ± 1.5	*p* = 0.0004

PD: Parkinson’s disease; HC: healthy controls; MV: mesencephalon volume.

## Supplementary Materials

The following figure is available online at https://www.mdpi.com/2076-3425/9/12/335/s1, Figure S1: Segmented and reconstructed live picture of midbrain with delineated substantia nigra.
