# Supplementary figures and images for: Semi-Automatic Signature-Based Segmentation Method for Quantification of Neuromelanin in Substantia Nigra
Source: Brain Sci. 2019 Nov 22;9(12):335. doi: 10.3390/brainsci9120335 (PMC6956028; doi:10.3390/brainsci9120335)

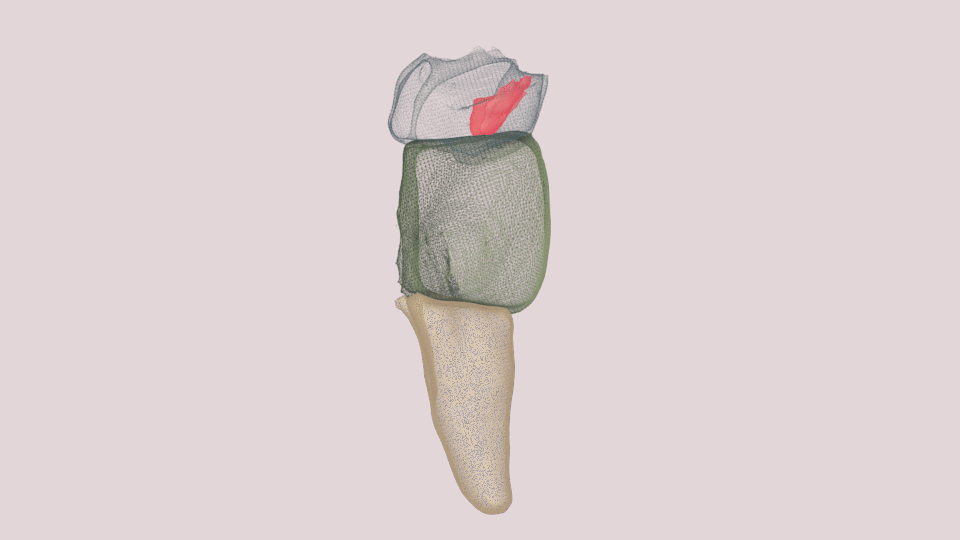

Supplement: Supplementary file 1 [file brainsci-09-00335-s001.zip › brainsci-636799-supplementary.gif]
